# Brain Vital Signs Detect Cognitive Improvements During Combined Physical Therapy and Neuromodulation in Rehabilitation From Severe Traumatic Brain Injury: A Case Report

**DOI:** 10.3389/fnhum.2020.00347

**Published:** 2020-09-10

**Authors:** Shaun D. Fickling, Trevor Greene, Debbie Greene, Zack Frehlick, Natasha Campbell, Tori Etheridge, Christopher J. Smith, Fabio Bollinger, Yuri Danilov, Rowena Rizzotti, Ashley C. Livingstone, Bimal Lakhani, Ryan C. N. D’Arcy

**Affiliations:** ^1^Centre for Neurology Studies, HealthTech Connex Inc., Vancouver, BC, Canada; ^2^BrainNET, Health and Technology District, Vancouver, BC, Canada; ^3^Applied Sciences and Sciences, Simon Fraser University, Vancouver, BC, Canada; ^4^Department of Kinesiology, University of Wisconsin-Madison, Madison, AL, United States; ^5^Pavlov Institute of Physiology, Russian Academy of Sciences, Saint Petersburg, Russia; ^6^Centre of Excellence in Mental and Physical Rehabilitation, Legion Veteran’s Village, Surrey, BC, Canada; ^7^Centre for Brain Health (Radiology), University of British Columbia, Vancouver, BC, Canada

**Keywords:** traumatic brain injury (TBI), translingual neurostimulation (TLNS), electroencephalography (EEG), brain vital signs, neuroplasticity, post-traumatic stress disorder (PTSD), case report

## Abstract

Using a longitudinal case study design, we have tracked the recovery of motor function following severe traumatic brain injury (TBI) through a multimodal neuroimaging approach. In 2006, Canadian Soldier Captain (retired) Trevor Greene (TG) was attacked with an axe to the head while on tour in Afghanistan. TG continues intensive daily rehabilitation, which recently included the integration of physical therapy (PT) with neuromodulation using translingual neurostimulation (TLNS) to facilitate neuroplasticity. Recent findings with PT + TLNS demonstrated that recovery of motor function occurred beyond conventional time limits, currently extending past 14-years post-injury. To investigate whether PT + TLNS similarly resulted in associated cognitive function improvements, we examined event-related potentials (ERPs) with the brain vital signs framework. In parallel with motor function improvements, brain vital signs detected significant increases in basic attention (as measured by P300 response amplitude) and cognitive processing (as measured by contextual N400 response amplitude). These objective cognitive improvements corresponded with TG’s self-reported improvements, including a noteworthy and consistent reduction in ongoing symptoms of post-traumatic stress disorder (PTSD). The findings provide valuable insight into the potential importance of non-invasive neuromodulation in cognitive rehabilitation, in addition to initial indications for physical rehabilitation.

## Introduction

Rehabilitation outcomes following traumatic brain injury (TBI) are often focused on the improvement of motor function to reduce the impacts of long-term disability (Langlois et al., 2006; Strong et al., 2007). Consequently, physical therapy (PT) can be an early adopter in the integration of novel neurotechnologies as adjuncts to best-practice clinical therapies. In particular, it is possible to monitor progress through functional neuroimaging (Gattu et al., 2016; Epps et al., 2019) and stimulate new progress through neuromodulation to promote enhanced recovery through neuroplasticity (Li et al., 2015; Danilov and Paltin, 2018). Using a longitudinal case study design, we have previously utilized multimodal functional imaging, including functional magnetic resonance imaging (fMRI), magnetoencephalography (MEG), and electroencephalography (EEG) to track the neurophysiological recovery of motor function (D’Arcy et al., 2016, 2020). In the most recent investigation, this approach was expanded to combine PT with neuromodulation using translingual neurostimulation (TLNS) to facilitate further recovery through facilitated neuroplasticity (D’Arcy et al., 2020).

Methodologically, TLNS can be done through the Portable Neuromodulation Stimulator (PoNS^®^), a Class II Health Canada approved medical device that applies sequenced, non-invasive electrical stimulation to the tongue (Helius Medical Technologies, Newtown, PA, USA). The PoNS^®^ delivers electrical stimulation through the tongue to the brain *via* the trigeminal and facial cranial nerves (CNV and CN-VII, respectively). The stimulation is hypothesized to modulate global brain function through the bottom-up brainstem and cerebellar pathways, augmenting neuroplasticity (Herrick and Keifer, 1998; Buisseret-Delmas et al., 1999; Marano et al., 2005; Wildenberg et al., 2011; Frehlick et al., 2019). Prolonged stimulation in combination with PT has generated a range of improvements, including improved gait and balance in individuals who have survived TBI (Leonard et al., 2017; Bastani et al., 2018; Danilov and Paltin, 2018; Tyler et al., 2019; Ptito et al., 2020).

In early trials, participants with mild-to-moderate traumatic brain injury (mmTBI) also reported incidental cognitive improvements (Danilov et al., 2015), suggesting that adaptive changes extend beyond sensorimotor functions (Danilov and Paltin, 2018). We recently used high-density EEG to confirm that a single 20-min session of TLNS stimulation significantly increased alpha and theta frequencies as well as attention microstate activity in healthy individuals (Frehlick et al., 2019). In a recent follow-up study (Smith et al., 2020), TLNS also significantly improved cognitive vigilance in healthy individuals measured using the brain vital signs framework.

The brain vital signs framework (Ghosh Hajra et al., 2016, 2018a; Fickling et al., 2019) provides a rapid evaluation of evoked brain responses through quantified EEG, extracted as event-related potentials (ERPs). ERPs provide an objective, physiological measure of cognitive function (Gawryluk and D’Arcy, 2010; Luck, 2014). The brain vital signs framework comprises a portable EEG system with a 6-min, automated assessment of three well-established sensation-to-cognition target ERP responses: (1) the N100 (Auditory sensation, Davis, 1939); (2) the P300 (Basic attention, Sutton et al., 1967); and (3) the Semantic N400 [Cognitive processing (Kutas and Hillyard, 1980)]. Ghosh Hajra et al. (2018b) subsequently demonstrated a comparable N400 response using common clinical contextual orientation questions frequently employed at the point-of-care (e.g., routine clinical cognitive screening such as knowing the day of the week). All three ERP types can be elicited from an auditory stimulation sequence comprised of tones and spoken word pairs. Response amplitude (microvolts) and latency (milliseconds) measures can be extracted for each of the three ERPs for a total of six measures (Ghosh Hajra et al., 2016). These measures can also be converted into standardized brain scores for intuitive interpretation within a radar plot format to visualize an overall cognitive function and subsequent change over time (Fickling et al., 2019). The brain’s vital signs framework has been successfully shown to be sensitive to cognitive changes in both healthy aging and brain injury (Ghosh Hajra et al., 2016; Fickling et al., 2019).

Accordingly, within the longitudinal case study of motor function (D’Arcy et al., 2020), we investigated whether PT + TLNS would also lead to detectable changes in cognitive processing using the brain vital signs framework. Based on the evidence to-date, we hypothesized that TLNS would significantly increase brain vital signs measures of attention and cognitive processing as a function of global neuroplasticity improvements.

## Case Description

Research ethics approval was obtained from the Research Ethics Boards of Simon Fraser University, Fraser Health Authority, and the National Research Council of Canada. As co-investigators and co-authors, Captain (retired) Trevor Greene (TG) and his wife Debbie Greene (DG) continue as collaborators in a combined PT/functional neuroimaging longitudinal case study that began in 2010. D’Arcy et al. (2016) provided a detailed case description. In summary, TG was 41-years-old, right-handed, and university-educated when he was on tour in Afghanistan in 2006. He was leading a meeting with Shinkay village elders on March 4, 2006, to help provide access to basic needs. He and the other soldiers had removed their helmets and laid down their weapons as a gesture of respect. At this point, a 16-year-old male pulled out an axe and swung it with two hands from above his head into the top of TG’s skull. The act signaled a Taliban attack.

The axe blow resulted in a severe open TBI with a deep penetrating injury along the midsagittal plane, extending from the frontal to the parietal lobes, with greater right frontal and left parietal damage relative to the sagittal suture. Primary motor, premotor, primary somatosensory, and superior parietal areas were affected. The depth of the injury extended from the cortex inferiorly to the lateral ventricle, affected anterior cingulate gyri, corpus callosum (body and genu), and surrounding white matter tissue.

Immediately afterward, TG underwent emergency care and was medevacked to Kandahar Airfield, where he was transferred to Germany for neurosurgical treatment and induced into a medical coma at the US Army Landstuhl Regional Medical Centre. TG was subsequently transported back to Vancouver General Hospital (Vancouver, BC, Canada), with an initial prognosis of permanent vegetative state. During acute care, he emerged from the coma and gradually recovered full consciousness over the following 18-months. He was then admitted to the Halvar Jonson Centre for Brain Injury Centre (Alberta, Canada) for a 14-month intensive rehabilitation program.

Since then, TG has managed severe physical movement disabilities through intensive daily rehabilitation. He has returned to his career as a journalist/writer after retiring from active duty, is a published author of several non-fiction books, speaker (including TEDx), and holds honorary doctorates along with other notable distinctions. He has actively engaged in cognitive training while managing on-going post-traumatic stress disorder (PTSD). For TG, on-going PTSD challenges include night terrors and being unable to sit with his back to a door. For a full description of TG and DG’s rehabilitation journal, see their book entitled: “March Forth: An Inspiring True Story of a Canadian Soldier’s Journey of Love, Hope, and Survival” (Greene and Greene, 2012). TG’s rehabilitation objectives continue to focus on recovering walking abilities along with all other related impacts. As a former elite rower, he applies intensive daily training together with mental imagery to continue to push the limits of his recovery.

The objective of the longitudinal case study has been to monitor TG’s recovery using multimodal functional neuroimaging (i.e., fMRI, MEG, and EEG). In the first phase (D’Arcy et al., 2016), fMRI was used four times a year over 3 years (12 times total) to monitor motor activation recovery. In parallel to clinical measures of movement recovery, a significant 5-fold increase in lower limb motor activation was observed. Of note, TG also showed significantly higher mental imagery activity (imagined rowing) relative to a matched control in the same active regions. The findings demonstrated neuroplasticity-related recovery well beyond conventional limits of 6-months to 1 year (6+ years at the time of the study).

The second phase incorporated assistive device technologies following an extended plateau in rehabilitation progress (D’Arcy et al., 2020). Following various assistive device trial evaluations, the study phase began in 2018 with the specific goal of investigating whether non-invasive neuromodulation, when paired with continuing physical rehabilitation, could help overcome the plateau of the recovery beyond 12-years post-injury. TLNS through the PoNS^®^ was highlighted in the book *The Brain That Changes Itself* (Doidge, 2007). To evaluate whether PT + TLNS could facilitate further recovery, we collected clinical, EEG, and MEG data during a 1-year PT only plateau period (i.e., baseline) and a 14-week PT + TLNS period (i.e., treatment). In a parallel study of motor function, PT + TLNS treatment led to clinically significant motor ability improvements with corresponding significant changes in both EEG and MEG activation suggesting network-level neuroplasticity effects in motor control function (D’Arcy et al., 2020). The present study focuses on the associated cognitive changes.

## Clinical Timeline and Methods

### Clinical Timeline

**Figure 1:**
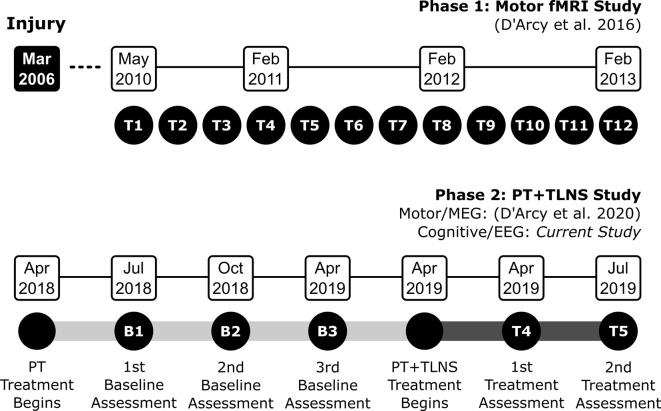
Overview of the experimental design and clinical timelines for the overall and current studies (B: Baseline; and T: Treatment).

D’Arcy et al. (2020) provided details of the clinical timeline for the PT + TLNS study. Briefly, TG underwent intensive PT-only through 2018 to establish a baseline and then began the 14-week PT + TLNS treatment phase. The PT + TLNS treatment phase was adapted from prior TLNS clinical trial methodology for TBI (Tyler et al., 2019; Ptito et al., 2020). Brain vital signs data were acquired in parallel for the three baseline time points (B1, B2, B3) and two treatment time points (T4 and T5). Figure 1 provides an overview of the clinical timeline.

### Methods

#### EEG Data Collection

EEG data were recorded using a portable 32-channel g.Nautilus system (g.tec Medical Engineering, Graz, Austria) with 10–20 electrode placement. gGAMMAsys gel was applied to each electrode location to maintain appropriate skin-electrode impedance. A reference electrode was attached to the right earlobe. To reduce motor and ocular artifacts, TG was instructed to sit motionlessly, and maintain visual fixation on a crosshair positioned at eye-level 2 m away. Two runs of the brain’s vital signs auditory stimulus sequence were collected at each time point.

#### EEG Data Processing

Data were processed in MATLAB (Mathworks, USA). A fourth-order Butterworth filter (1–20 Hz) and a custom Notch filter (60 Hz) were applied to the raw EEG data. Adaptive filtering was implemented to correct for ocular artifacts as recorded by the Fp1 and Fp2 electrodes (He et al., 2004). EEG from central (CZ, FC1, FC2, C3, C4), left temporal (F7, FC5, T7, P7, CP5), and right temporal (F8, FC6, T8, CP6, P8) channels were then pooled to increase SNR. ERPs were derived from these representative channels through standard methods of segmentation (range: −100 ms pre-stimulus to 900 ms post-stimulus), baseline correction, and conditional averaging (Luck, 2014). A wavelet filter was used to denoise the individual ERP epochs before final averaging (Quiroga and Garcia, 2003). Only the first run of each collection was used for the analysis, except in one instance where the ERP timing data were missing, and the second scan was used.

For each channel group, time-point, and stimulus type, epochs are randomly selected (with replacement) and averaged to form an ERP on which peaks are identified. ERP peaks were selected with an automated algorithm that chooses local maxima/minima within expected polarities and temporal ranges for the N100 (minima: 80–200 ms), P300 (maxima: 200–500 ms), and N400 (minima: 300–700 ms). Semantic and context N400 peaks were assessed on the difference waves between congruent/incongruent and relevant/irrelevant stimuli, respectively. After peak identification, each ERP feature is evaluated for both peak latency and baseline-adjusted amplitude. This is repeated 1,000 times to create a distribution of peak features for each condition. The mean and standard errors of these distributions better represent the confidence of the true mean of the underlying ERP (Di Nocera and Ferlazzo, 2000; Wilcox, 2012). The contextual N400 was selected over the semantic N400 for subsequent analysis due to a greater difference between stimulus conditions (see description of context N400 above).

#### Statistical Analysis

A non-parametric percentile-bootstrapping approach was then taken to obtain single-subject statistical comparisons of ERP features over the different time points (Wilcox, 2012). For each treatment time point, channel group, and stimulus type the ERP epochs for that assessment time were pooled with the ERP epochs from the three baseline assessments. Bootstrap resampling (5,000 iterations, with replacement) with ERP feature selection on the pooled data return a null distribution by which the mean ERP feature from just that treatment time point can be compared. If this falls above the 95th percentile of the null distribution, then the null hypothesis is rejected and the treatment measurement is significantly greater than the baseline. The exact percentile then represents the single-tailed *p*-value. All *p*-values were adjusted for multiple comparisons across individual ERP features (i.e., six comparisons) using the Benjamini-Hochberg False-Discovery Rate (FDR) procedure (Benjamini and Hochberg, 1995; Yekutieli and Benjamini, 1999).

Finally, mean ERP amplitudes and latencies were transformed into standardized scores from 0 to 100. The use of radar plots for the display of these scores allows for directional changes in multiple ERP metrics to be displayed on the same radial axis for simultaneous interpretation. These changes can create different profiles for different conditions. For example, prior research in brain vital signs has demonstrated different profiles for concussed and non-concussed athletes (Fickling et al., 2019).

## Clinical Results and Outcomes

The percentile bootstrapping analysis showed significant increases between the first treatment assessment (T4) and baseline (B1–3) in N100 amplitude (Central: *p* = 0.0198, *p*_adj_ = 0.0297; Right Temporal: *p* = 0.0186, *p*_adj_ = 0.0297). By the second treatment assessment (T5), significant increases from baseline were observed in N100 amplitude (Central: *p* = 0.0196, *p*_adj_ = 0.0297; Right Temporal: *p* = 0.0162, *p*_adj_ = 0.0297), P300 amplitude (Central: *p* = 0.0042, *p*_adj_ = 0.0252; Right Temporal: *p* = 0.0086, *p*_adj_ = 0.0258), and N400 amplitude (Right Temporal: *p* = 0.0062, *p*_adj_ = 0.0374). No significant effects were observed in ERP latency metrics or in the Left Temporal group of electrodes.

**Figure 2:**
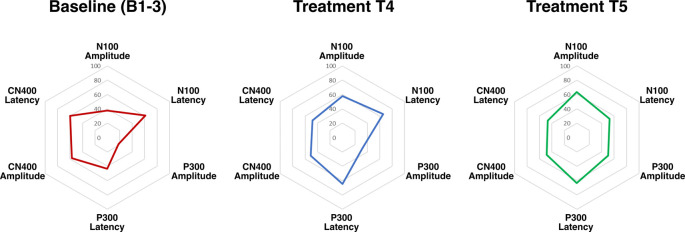
Radar plots of brain vital signs amplitudes and latencies from central electrodes for all three event-related potentials (ERPs) transformed into standardized scores (6 total). Increased ERP amplitudes equate to higher scores, and decreased ERP latencies equate to higher scores.

**Figure 3:**
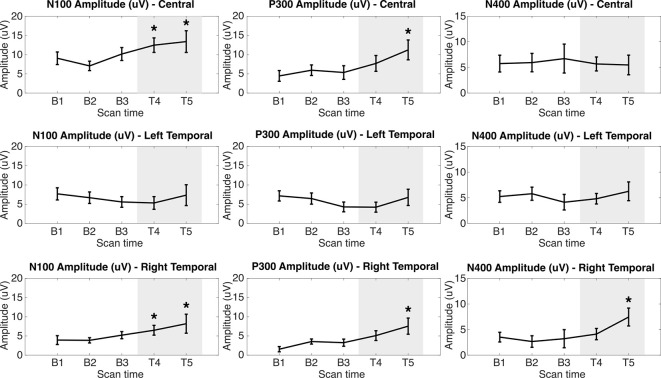
N100, P300, and N400 ERP amplitudes in microvolts at each assessment across Central Left Temporal, and Right Temporal electrode groups. Gray area indicates PT + TLNS treatment. *Indicates *p* < 0.05 relative to pooled-baseline [False-Discovery Rate (FDR) corrected].

Radar plots of standardized brain vital signs for the average baseline assessments and each treatment assessment during the TLNS treatment period are shown in Figure 2. For simplicity, only the central channel group are shown. Line graphs (mean ± standard error) for the longitudinal ERP changes in N100, P300, and N400 amplitudes (in μV) across all three electrode groups and all five time-points are presented in Figure 3. Corresponding latency changes over time are included in Supplemental Data.

Also, TG reported a considerable reduction in PTSD symptoms during and after the TLNS treatment, specifically a reduction in both frequency and intensity of night terrors.

## Discussion

### Main Findings

The findings support the hypothesis that PT + TLNS led to increased cognitive processing relative to the preceding PT-only baseline. Cognitive processing was objectively evaluated using well-established neurophysiological ERP responses within the brain vital signs framework (Figure 2). Specifically, PT + TLNS treatment showed significant increases in markers of Auditory sensation (N100), Basic attention (P300), and Cognitive processing (N400) amplitudes (Figure 3). The strength of the findings in this case report is derived from the demonstration that PT + TLNS rehabilitation for motor-control led to associated improvements in cognitive processing as a function of broader system-level neuroplasticity effects. The general increase in the area of the radar plot from baseline through to the second treatment is driven by the significant changes in N100, P300, and N400 amplitudes (Figures 2, 3).

### Global Neuromodulation Effects

Taken together with prior evidence of TLNS-related impacts on cognitive processing, it is noteworthy that TLNS has previously increased attention and cognitive semantic processing in healthy individuals (Frehlick et al., 2019; Smith et al., 2020). The current findings add to this with comparable evidence in recovery from severe TBI, further reinforcing the concept that TLNS has extended system-level neuroplasticity effects. TLNS is known to modulate sensorimotor and vestibular functions and is hypothesized to modulate multiple physiological processes *via* bottom-up neuromodulation of global brain function to augment neuroplasticity (Herrick and Keifer, 1998; Buisseret-Delmas et al., 1999; Marano et al., 2005; Wildenberg et al., 2010, 2011). Current models emerging from other neuromodulation technologies, such as deep brain stimulation, have increasingly interpreted the effects as being comparable to neuromodulation through a sensory organ, with access to the same pathways and translated in similar ways as natural environmental stimuli (Black and Rogers, 2020). This concept is consistent with exercise-induced neuroplasticity effects cascading across the molecular, cellular, structural, functional, and behavioral levels (El-Sayes et al., 2019) along with demonstrated neuroprotective effects of trigeminal nerve stimulation in TBI (Chiluwal et al., 2017).

### Attention and PTSD

The P300 is an indicator of the engagement of attentional resources to the stimuli, particularly related to the awareness of an individual’s surrounding environment (Polich, 2007). As such, the P300 ERP is one of the most widely studied evoked potentials in PTSD. Typically, the literature indicates abnormalities in information processing in individuals diagnosed with PTSD and that differences in ERP features are correlated with the severity of the illness (Javanbakht et al., 2011). Studies have shown decreased P300 amplitudes in groups with PTSD relative to control (Boudarene and Timsit-Berthier, 1997; Felmingham et al., 2002; Araki et al., 2005). It is thought that this is due to individuals with PTSD allocating attentional resources to persisting cues of trauma instead of the auditory stimulus from the ERP assessment (Javanbakht et al., 2011). This is further exacerbated in cases such as TG where attentional resources are also impacted through injury. Therefore, the significant increase in P300 amplitude observed during the TLNS treatment likely represents specific improvements in attention from injury recovery as well as general improvements in PTSD and subsequent changes in attentional resource allocation.

### Caveats

Some caveats are worth highlighting: (1) ongoing longitudinal monitoring of TG will be important to continue to characterize his recovery over time; where (2) study designs that specifically employ quantitative measures of PTSD (such as DSM-5) should be implemented. (3) TG represents a unique case and the degree to which these results generalize needs to be examined; therefore (4) the ability of PT + TLNS to improve both cognitive function and PTSD requires replication in larger samples of patients with TBI and/or psychological trauma.

### Key Takeaway

TG’s is a unique case that adds considerable value to the body of knowledge regarding the possibilities of recovery after major TBI. The key takeaway underscores the role of non-invasive neuromodulation in harnessing the positive impacts of neuroplasticity.

## Conclusion

This case study shows the ongoing recovery of TG, who received a severe open TBI in 2006, as measured by the brain’s vital signs evoked potential framework. When undergoing PT + TLNS, compared to PT alone, TG showed significant increases in markers of basic attention (as measured by P300 response amplitude) and cognitive processing (as measured by contextual N400 response amplitude). These cognitive improvements coincided with objective improvements in motor function and TG’s self-reported improvements including a noteworthy and consistent reduction in ongoing PTSD symptoms. The findings provide valuable insight into the potential importance of non-invasive translingual neurostimulation in cognitive rehabilitation for neurological conditions, in addition to initial indications for physical rehabilitation.

## Patient Perspective

TG and DG are uniquely able to provide patient, family, investigator, and coauthor perspectives. Having passed 14-years post-injury, they confirm that intensive daily rehabilitation alone has involved plateaus in progress and that TLNS has been instrumental in restarting their recovery journey. TG continues to confirm on-going progress, including a continued reduction in PTSD symptoms. Following the initial 14-week PT + TLNS trial, DG stated *“I got my superman back”*.

## Data Availability Statement

The datasets presented in this article are not readily available because of patient-related sensitivities. Requests to access the datasets should be directed to RD’A: ryan@healthtechconnex.com.

## Ethics Statement

The studies involving human participants were reviewed and approved by Simon Fraser University Research Ethics Board, Fraser Health Research Board, and National Research Council Research Ethics Board. The patients/participants provided their written informed consent to participate in this study. Written informed consent was obtained from the individual(s) for the publication of any potentially identifiable images or data included in this article.

## Author Contributions

All authors: conceptualization and study design, analysis planning, analysis outcome verification, result interpretation, critical editing and approval of submission. SF, NC, and RD’A: literature search. SF and RD’A: data analysis and manuscript preparation. SF, ZF, CS, TG, DG, and BL: data collection. SF, NC, BL, and RD’A: result presentation. All authors contributed to the article and approved the submitted version.

## Conflict of Interest

Authors SF, ZF, NC, TE, CS, FB, RR, AL, BL, and RD’A were employed by the company HealthTech Connex Inc. The remaining authors declare that the research was conducted in the absence of any commercial or financial relationships that could be construed as a potential conflict of interest.
