# Supplementary material for: Brain Vital Signs Detect Cognitive Improvements During Combined Physical Therapy and Neuromodulation in Rehabilitation From Severe Traumatic Brain Injury: A Case Report
Source: Front Hum Neurosci. 2020 Sep 10;14:347. doi: 10.3389/fnhum.2020.00347 (PMC7513585; doi:10.3389/fnhum.2020.00347)
Supplement: Supplementary file 1 [file Data_Sheet_1.docx]

**
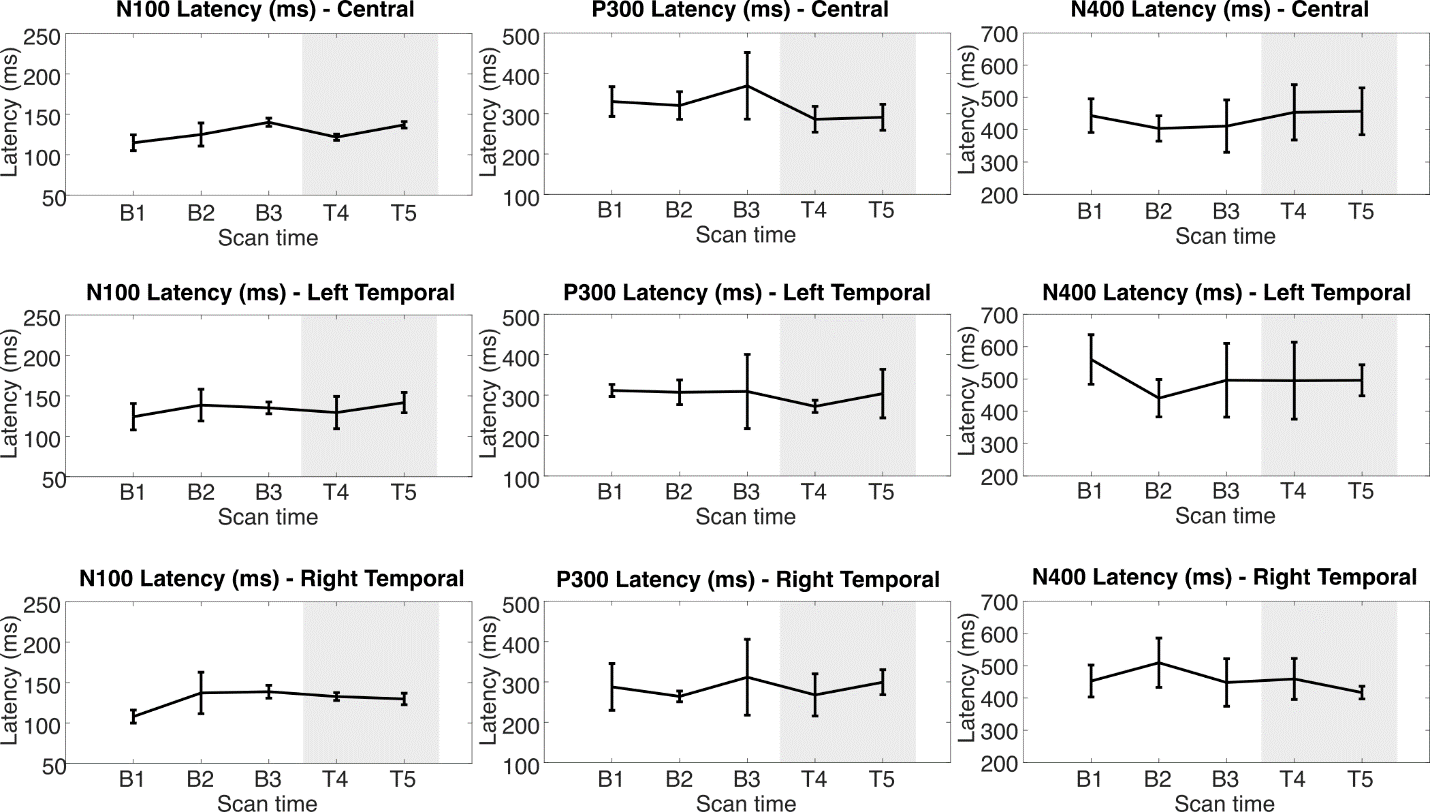
**

Supplemental Figure 1: N100, P300, and N400 ERP latencies in milliseconds at each assessment across Central, Left Temporal, and Right Temporal electrode groups. Grey area indicates PT+TLNS treatment. * indicates p<0.05 relative to pooled-baseline (FDR corrected).
